# Watchful Expectant Management of a Giant Placental Chorioangioma With Favorable Maternal and Fetal Outcomes: A Case Report

**DOI:** 10.7759/cureus.39496

**Published:** 2023-05-25

**Authors:** Rekha Patidar, Krishi Gowdra Revannasiddappa, Suvarna Mestha

**Affiliations:** 1 Obstetrics and Gynaecology, Zulekha Hospital, Dubai, ARE; 2 Obstetrics and Gynaecology, American Hospital Clinic, Dubai, ARE

**Keywords:** fetal anemia, doppler ultrasound, conservative management, polyhydramnios, chorioangioma

## Abstract

Placental chorioangioma is the most common benign tumor of the placenta. However, clinically evident chorioangiomas are less common with an incidence ranging between 1:3,500 and 1:9,000 births. In the majority of cases, it is small or microscopic and of no clinical significance. If it increases more than 5 cm in size, then it may be associated with serious maternal complications such as mirror syndrome, polyhydramnios, preeclampsia, antepartum hemorrhage, preterm labor and delivery, and postpartum hemorrhage, as well as fetal complications such as fetal anemia, fetal thrombocytopenia, fetal hydrops, intrauterine growth restriction, fetal demise, and neonatal death. Giant chorioangioma associated with polyhydramnios leads to high perinatal morbidity and mortality. Chorioangioma with complications before fetal viability needs interventions. Conservative management with close surveillance can be considered in selective cases. We report a case of giant placental chorioangioma complicated with severe polyhydramnios managed conservatively with favorable maternal and fetal outcomes.

## Introduction

Chorioangioma is a non-trophoblastic benign vascular tumor of the placenta arising from primitive chorionic mesenchyme. These tumors consist of multiple fetal capillaries supported by stroma and predominantly perfused by fetal circulation. The prevalence of chorioangiomas is about 1% [1]. Most chorioangiomas are small, asymptomatic, and generally of no clinical significance. On the other hand, chorioangiomas larger than 5 cm in diameter are rare, with their prevalence ranging from 1:9,000 to 1:50,000 [2]. The size and vascularity of chorioangioma are the main prognostic indicators of maternofetal outcomes [2]. Large chorioangiomas are associated with significant arteriovenous shunting within the placenta leading to fetal anemia, high-output cardiac failure, hydrops, intrauterine growth restriction, and stillbirths [3-6]. Placental sequestration within the mass can also cause fetal microangiopathic anemia and thrombocytopenia [3,7]. The transudate from the tumor vessels across the chorionic plate with increased fetal urine output associated with fetal hyperdynamic circulation caused either by shunting of blood to placental chorioangioma or fetal anemia leads to polyhydramnios, which, in turn, can cause maternal abdominal distention, preterm labor, abruptio placentae, and postpartum hemorrhage [8-10]. Large chorioangiomas causing maternofetal complications need interventions such as endoscopic surgical devascularization, alcoholic ablation, and interstitial laser coagulation [7]. Amnioreduction may provide temporary relief in severe polyhydramnios. Close monitoring with serial ultrasound and Doppler may allow conservative management with favorable outcomes in selected cases [11]. Here, we present a case of giant chorioangioma managed expectantly with favorable maternal and fetal outcomes.

## Case presentation

A 32-year-old, G2P0L0A1, pharmacist, from good socioeconomic status, presented in the antenatal clinic at five weeks of amenorrhea. Pregnancy was confirmed by ultrasound and folate supplementation was commenced. At six weeks of gestation, viable pregnancy was confirmed by ultrasound. She had a non-consanguineous marriage. At 12 weeks, booking antenatal investigations and a nuchal translucency scan were performed and were found to be normal. A double marker was not done as the patient declined.

**Figure 1:**
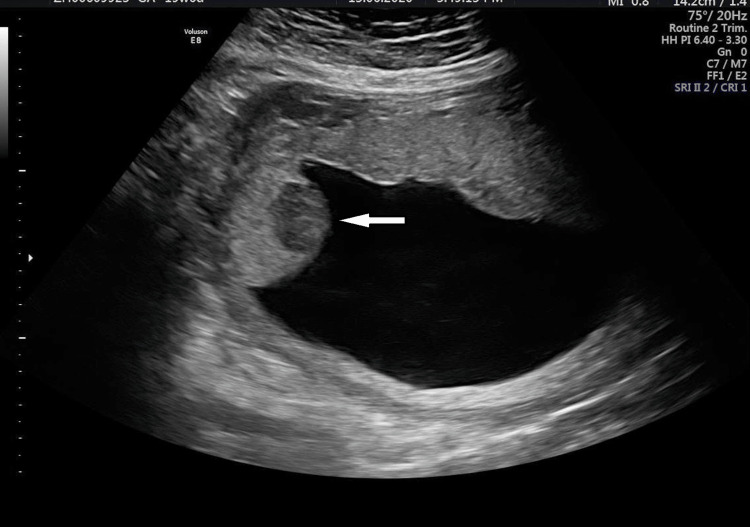
Ultrasound image at 20 weeks of gestation.

At 20 weeks of gestation, an anomaly scan revealed a single viable fetus with no obvious anomalies, but the placenta showed evidence of a hypoechoic lesion in the upper margin measuring 2 × 1.8 cm with minimal vascularity, suggestive of possible chorioangioma (Figure 1). The couple was counseled about her ultrasound report of chorioangioma, possible maternal and fetal complications associated with it, the need for close antenatal monitoring, possible interventions, and preterm delivery if needed.

**Figure 2:**
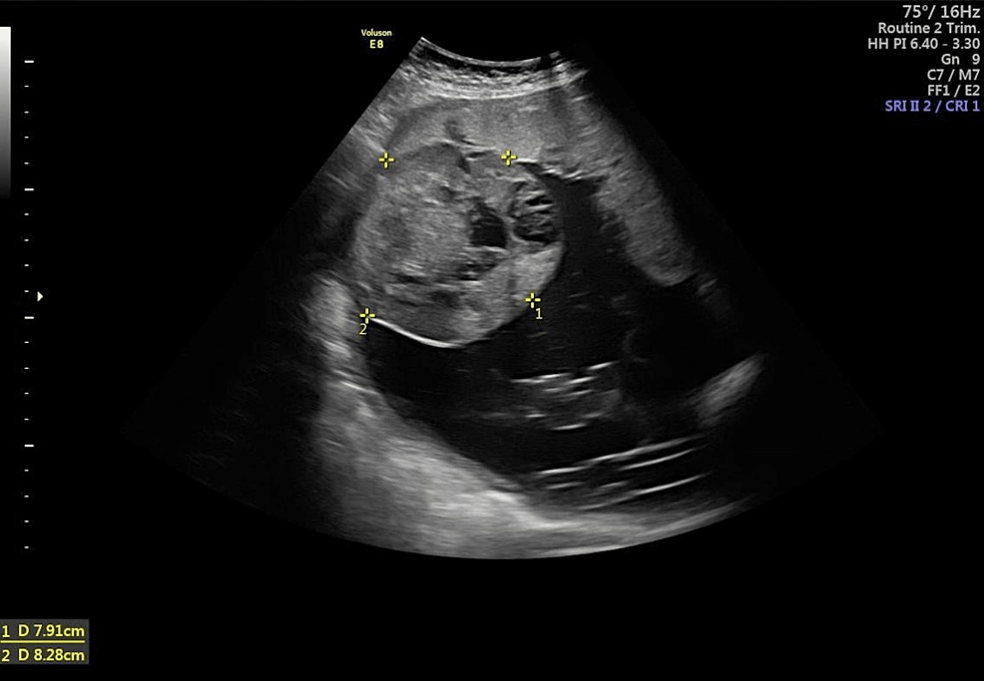
Ultrasound image at 31 weeks of gestation.

**Figure 3:**
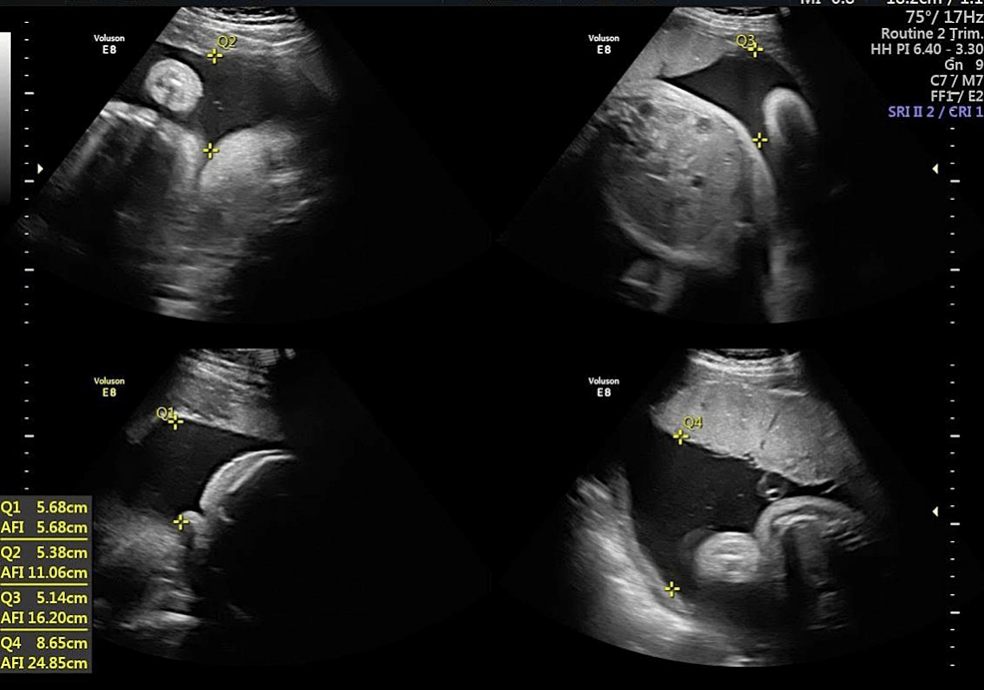
Ultrasound image at 31 weeks of gestation showing polyhydramnios.

A repeat scan performed at 27 weeks showed a single viable fetus of 28 weeks gestation with normal liquor volume. The placenta showed a well-circumscribed iso/hypoechoic lesion at the superior end of the placenta at the fundus protruding into the amniotic cavity measuring 6.2 × 6.1 cm with vascularity similar to the placenta and a few cystic spaces seen within it. Alpha-fetoprotein (244.3 IU/mL) and beta-human chorionic gonadotropin (44,297 mIU/mL) were found to be elevated. After radiological confirmation of the diagnosis of chorioangioma, a multidisciplinary team meeting involving a senior consultant obstetrician, radiologist, and neonatologist was held and a further plan of management was decided. At the 31-week antenatal check-up, her general condition was normal, but her abdomen was distended and fundal height corresponded to 34 weeks gestation. Ultrasound showed a further increase in the size of chorioangioma to 8 × 8.2 cm (Figure 2) and polyhydramnios (amniotic fluid volume of 24 cm) (Figure 3). Doppler parameters were normal, and the middle cerebral artery peak systolic velocity (MCA-PSV) was 41 cm/second.

**Figure 4:**
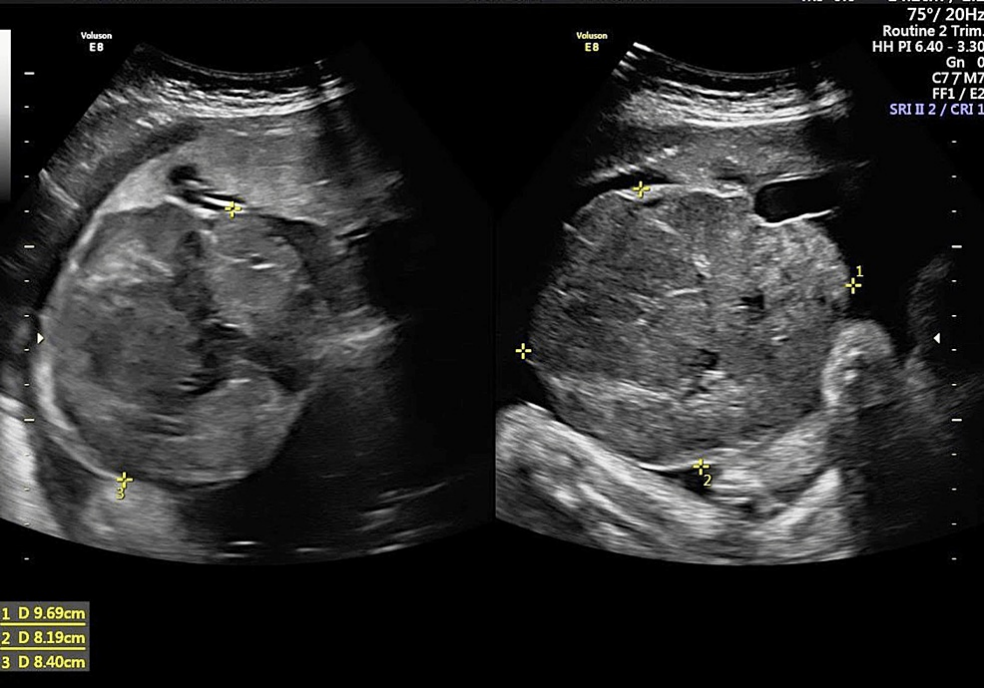
Ultrasound image at 33 weeks of gestation.

At 33 weeks, she complained of difficulty in moving and lying down due to abdominal distension. Ultrasound revealed an increased size of chorioangioma to 10 cm × 10 cm, amniotic fluid volume of 28 cm, normal fetal growth, and normal fetal Doppler (Figure 4). She was counseled about the possibility of preterm delivery, and prophylactic steroids to accelerate lung maturity were advised.

**Figure 5:**
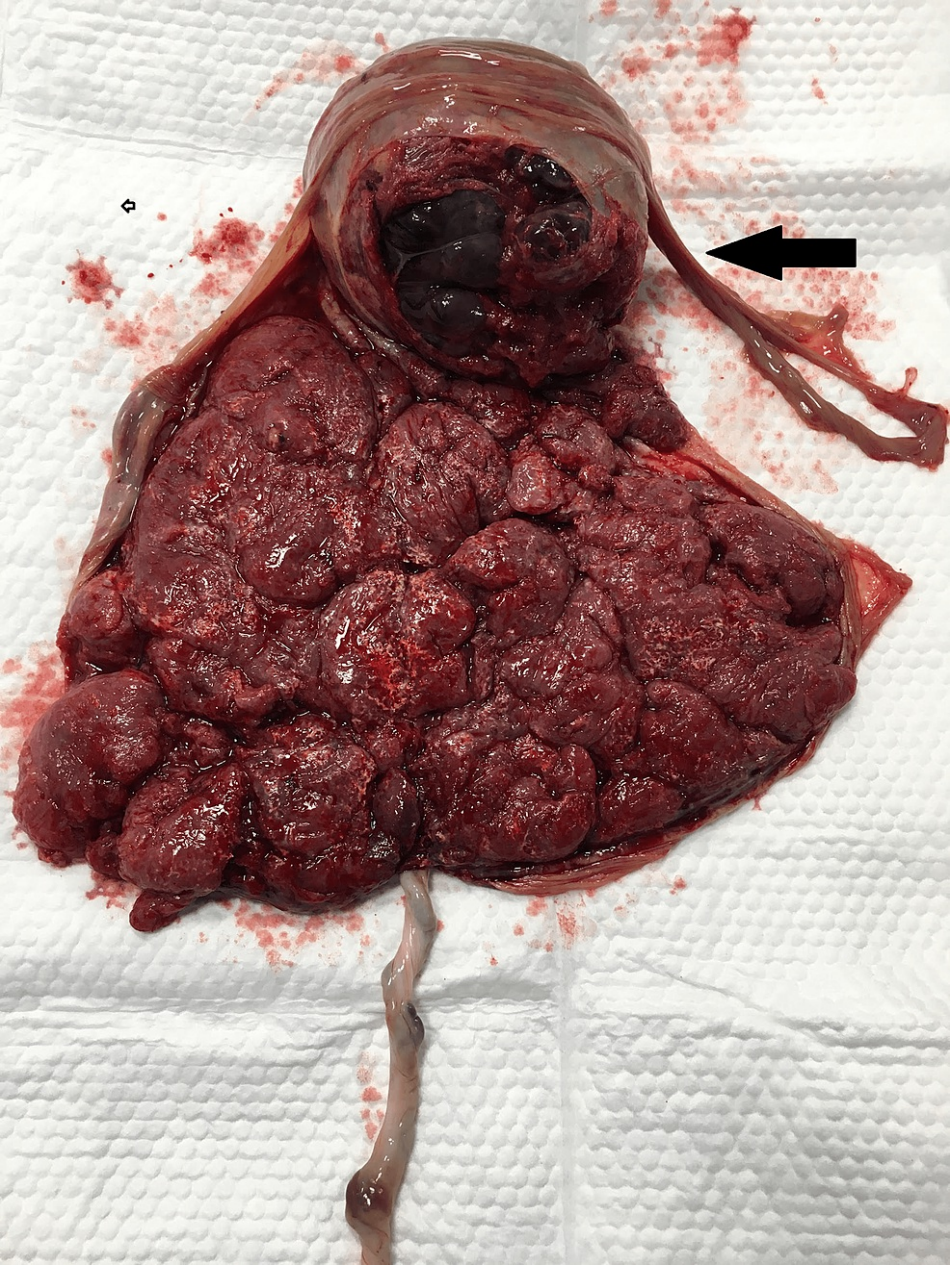
Gross specimen of the placenta with the chorioangioma - maternal surface.

**Figure 6:**
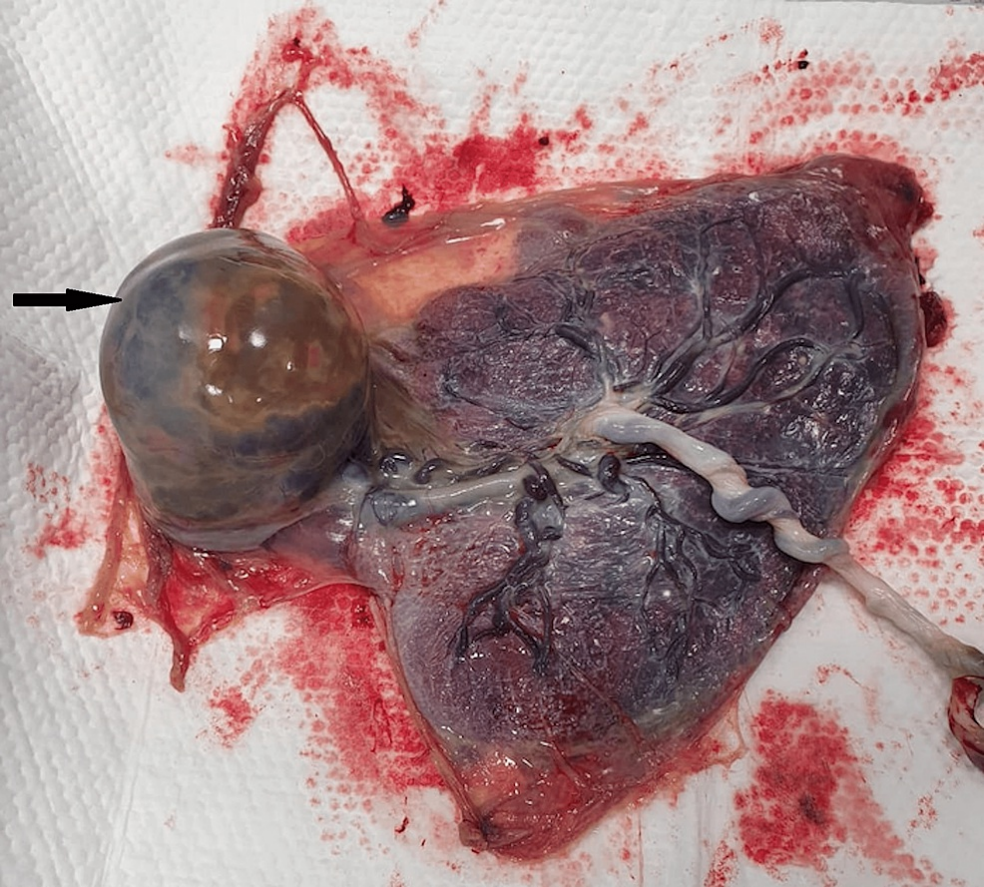
Gross specimen of the placenta with chorioangioma - fetal surface.

At 35 weeks of gestation, she presented with worsening abdominal distension, pain, and reduced fetal movements. She was admitted for observation. Cardiotochograph was reassuring with mild irregular uterine contractions. Ultrasound revealed a further increase in the size of chorioangioma to 12 cm × 10 cm, amniotic fluid volume of 40 cm, normal fetal growth, and normal fetal Doppler. In view of a persistent feeling of reduced fetal movements and persistent abdominal pain, the decision was taken for a cesarean section. The couple was counseled about the need for care in the neonatal intensive care unit (NICU), the risk of postpartum hemorrhage, and the interventions needed to manage it. A possible hysterectomy was also explained and consented to in case of intractable postpartum hemorrhage. A cesarean section was performed under spinal anesthesia when she delivered a live female baby with a birth weight of 2,455 g and APGAR scores of 6, 8, and 9 at one, five, and ten minutes. The liquor was grade 1 meconium stained and about 4 L in volume. Watchful expectancy was adopted for placental delivery. The placenta along with the chorioangioma delivered spontaneously weighing 1,400 gm, with the chorioangioma measuring 12 × 10 × 10 cm in size (Figures 5, 6).

There was no immediate postpartum hemorrhage, but in view of poor uterine tone and placental bed oozes, a Bakri balloon was inserted and inflated with 400 mL of normal saline and was removed after eight hours. Her postoperative recovery was unremarkable, and she was discharged on day three postoperatively.

**Figure 7:**
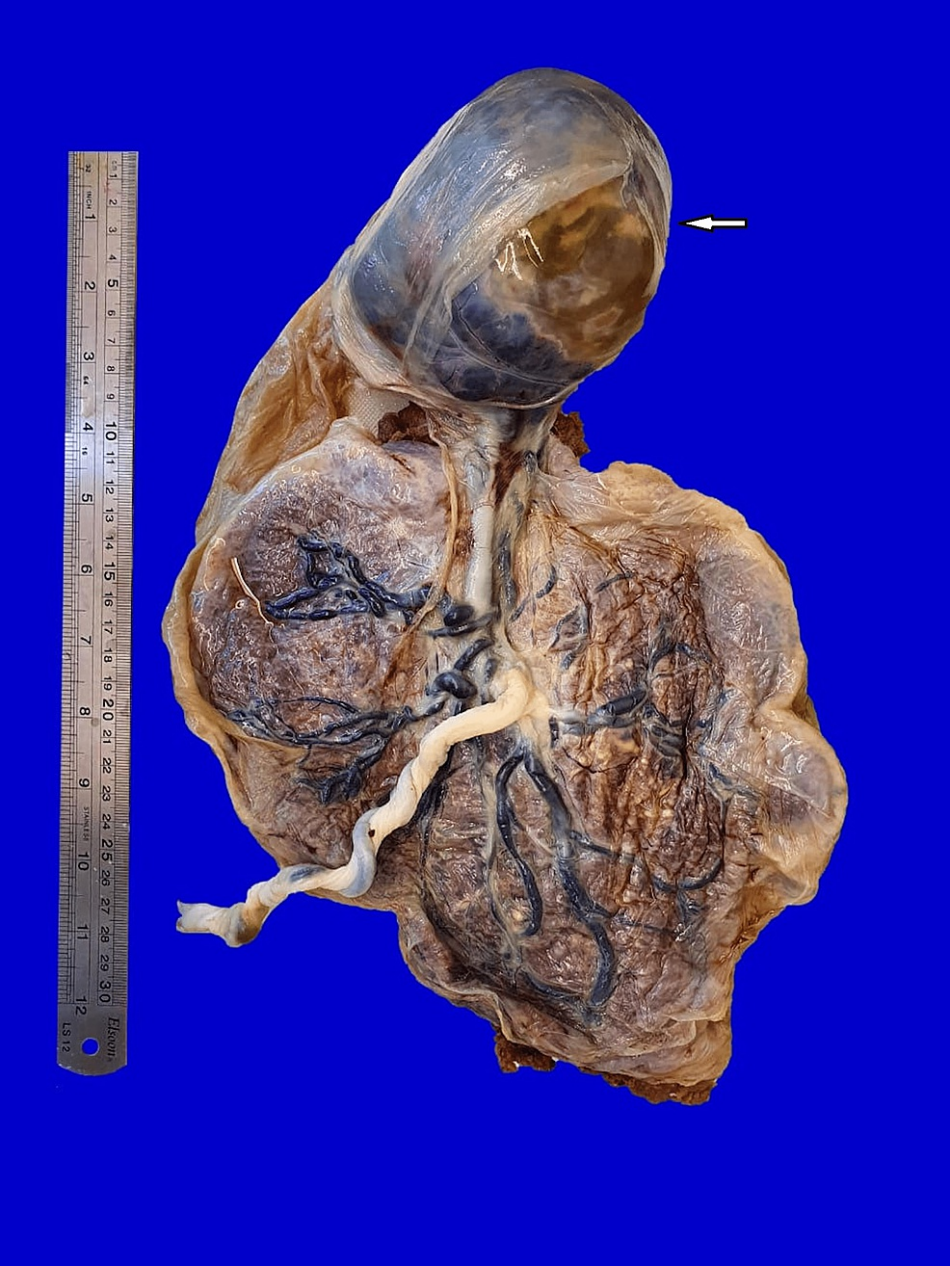
Histopathology - gross picture.

**Figure 8:**
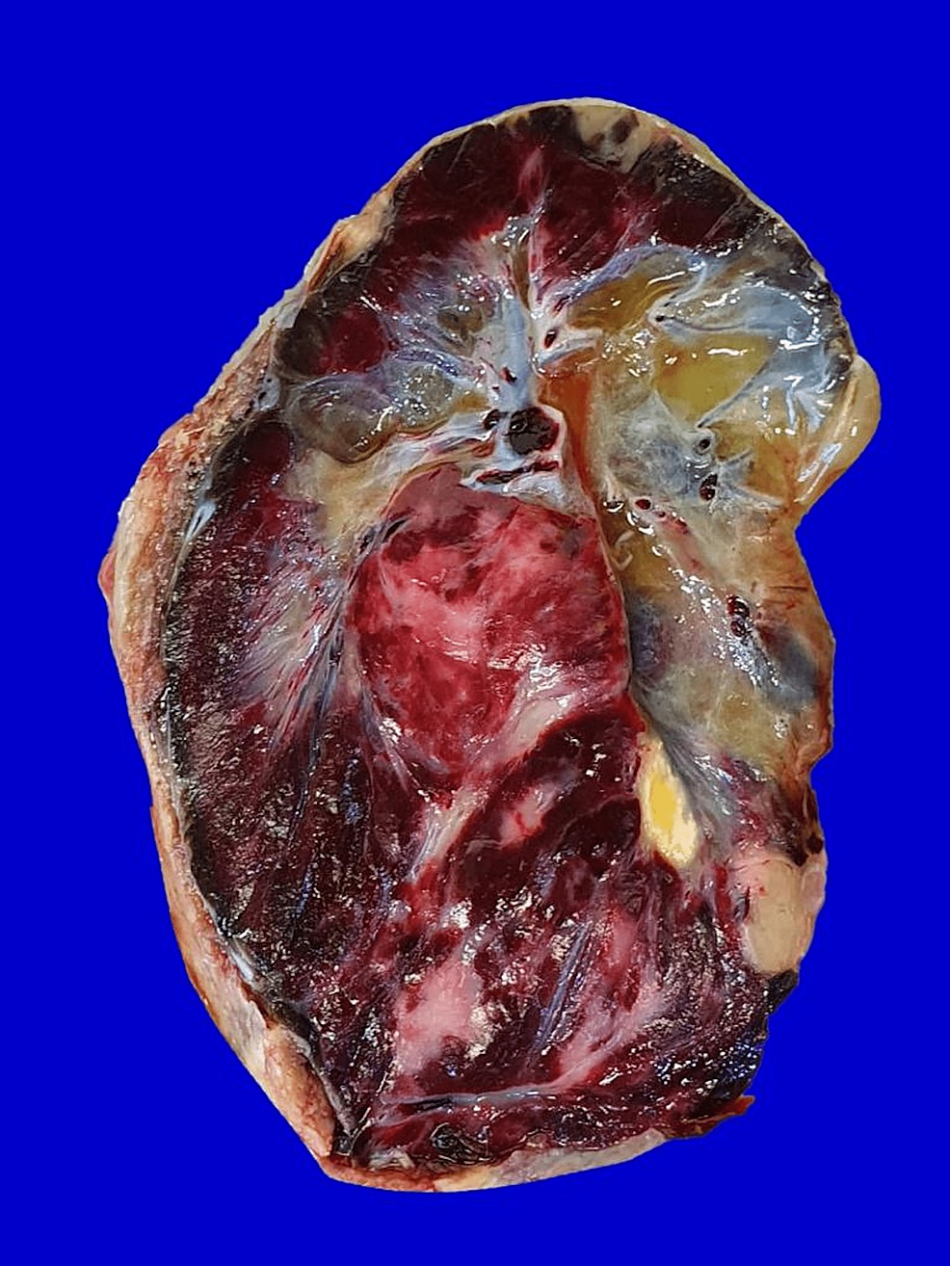
Gross picture of the cut surface of the chorioangioma - reddish solid cut surface with focal myxoid changes and yellowish areas.

**Figure 9:**
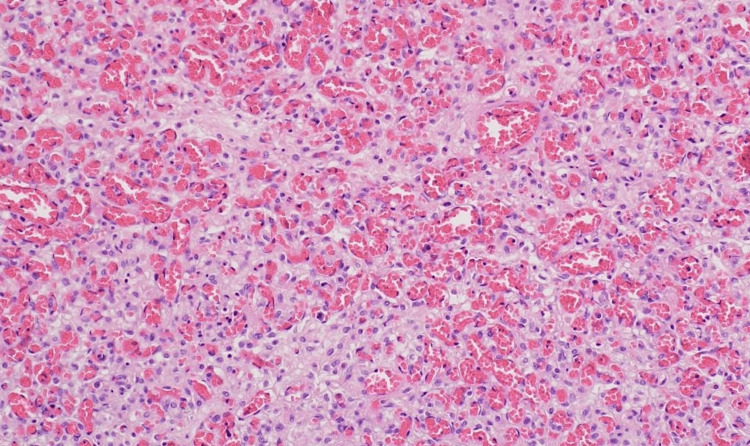
Histopathology - microscopic picture shows the proliferation of capillary-sized vessels lined by endothelial cells with pericytes and myofibroblastic stromal cells.

Histopathological evaluation of the placenta confirmed chorioangioma with no evidence of malignancy (Figures 7-9).

The baby was given supportive and respiratory care for 48 hours and then was discharged on day 7 of the NICU stay. Beta-human chorionic gonadotropin and alpha-feto protein levels returned to the completely normal range at 2 months postnatally. At 1.5 years of baby age, she had all normal neurobehavioral milestones.

## Discussion

Chorioangioma is the most frequent non-trophoblastic benign tumor of the placenta. Giant chorioangioma greater than 4 cm in diameter is rarely seen and is associated with complications, including polyhydramnios, fetal anemia, fetal cardiomegaly, hydrops, and increased perinatal morbidity and mortality [9]. No definitive etiology has been reported. Chorioangiomas are often associated with elderly primiparity, twin pregnancy, preeclampsia, and diabetes [4]. Chorioangioma can be diagnosed by ultrasound as a well-circumscribed, round, predominantly hypoechoic mass, often near to the cord insertion site along the fetal surface of the placenta [12]. They are known to protrude into the amniotic cavity. Chorioangioma can be differentiated from other lesions such as degenerated fibroid, placental hematoma, placental teratoma, and deceased twins by color Doppler. Magnetic resonance imaging (MRI) can also aid in diagnosis as T2-weighted MRI images are similar to hemangioma [2]. Grossly, chorioangioma is a well-circumscribed purplish red tumor with a fleshy, congested, red-to-tan cut surface [2,13]. Microscopically, these tumors demonstrate angiomatous and cellular matrix with degenerative changes of calcification [13]. In large tumors, hyaline and myxoid degeneration is noticed, as in our case. The presence of proliferative blood vessels in various stages of differentiation, surrounded by placental stroma, and the absence of villi differentiate chorioangioma from chorioangiosis and chorioangiomatosis [2,14]. No fixed management guidelines have been reported in the literature to date. They can be managed conservatively or by therapeutic surgical interventions such as the direct injection of various chemicals and laser coagulation of feeding vessels [3,14]. If signs of fetal cardiac failure/anemia are seen in early pregnancy, then the patient should be referred to the fetal medicine unit for interventions, but when they occur late in pregnancy, delivery is indicated. Polyhydramnios can be treated by therapeutic amniocentesis and maternal indomethacin therapy [9]. In our case, polyhydramnios without a concomitant increase in MCA-PSV supports the placental transudate hypothesis. We adopted a conservative approach as there were no fetal complications. In our case, severe polyhydramnios occurred in the third trimester around 33-35 weeks of gestation, hence, we administered prophylactic steroids for fetal lung maturation and considered the patient for delivery rather than therapeutic amniocentesis. When a conservative approach is adopted, patients should be fully counseled about complications and management plans and monitored more frequently with ultrasound scanning and fetal Doppler.

## Conclusions

A target scan at 20 weeks has made the early diagnosis of chorioangiomas possible. Further, serial antenatal scans and Doppler ultrasound play an important role in monitoring and deciding treatment modalities. Detailed serial scans and Doppler can identify early complications and help in timely interventions if required. Early-onset complications need a referral to a fetal medicine unit; however, late-onset complications can be managed with close monitoring and timely delivery. Further studies/research is required to formulate selection criteria for conservative and interventional management. Such patients should have institutional delivery where appropriate expertise and NICU facilities are available. Obstetricians should be able to anticipate, prevent, and treat antenatal and intrapartum complications.
